# Heterogeneity in Trajectories of Systolic Blood Pressure among Young Adults in Qingdao Port Cardiovascular Health Study

**DOI:** 10.5334/gh.764

**Published:** 2020-03-02

**Authors:** Haiqun Lin, Meiping Cui, Erica S. Spatz, Yongfei Wang, Jiapeng Lu, Jing Li, Shuxia Li, Chenxi Huang, Xiancheng Liu, Lixin Jiang, Harlan M. Krumholz, Xiao Xu

**Affiliations:** 1 Division of Nursing Science, School of Nursing, Rutgers Biomedical and Health Sciences, Newark, NJ, US; 2 Section of Cardiovascular Medicine, Qingdao Fuwai Hospital, Qingdao, CN; 3 Center for Outcome Research and Evaluation, Yale School of Medicine, New Haven, CT, US; 4 China Oxford Centre for International Health Research, National Center for Cardiovascular Diseases, Fuwai Hospital, CAMS, Beijing, CN; 5 Department of Obstetrics, Gynecology, and Reproductive Sciences, Yale School of Medicine, New Haven, CT, US

**Keywords:** systolic blood pressure, trajectories, young adults, mixture model

## Abstract

**Background:**

Although increased age is associated with higher systolic blood pressure (SBP) in general, there may be variation across individuals in how SBP changes over time. The goal of this paper is to identify heterogeneity in SBP trajectories among young adults with similar initial values and identify personal characteristics associated with different trajectory patterns. This may have important implications for prevention and prognosis.

**Methods:**

A cohort of 12,468 individuals aged 18–35 years in the Qingdao Port Cardiovascular Health Study in China was followed yearly during 2000–2011. Individuals were categorized into three strata according to their baseline SBP: ≤110 mmHg, 111–130 mmHg, and >130 mmHg. Within each stratum, group-based trajectory analyses were conducted to identify distinct SBP trajectory patterns, and their association with sociodemographic and baseline health characteristics was assessed by ordinal logistic regression.

**Results:**

Five distinct groups of individuals exhibiting divergent patterns of increasing, stable or decreasing SBP trends were identified within each stratum. This is a first report to identify a subgroup with decreasing trend in SBP. Individuals with more advanced age, having less than high school education, family history of cardiovascular diseases, greater body mass index, greater waist circumference, and hyperlipidemia at baseline were more likely to experience trajectories of higher SBP within each stratum.

**Conclusions:**

The diverging trajectories among young adults with similar initial SBP highlight the need for prevention and feasibility of effective blood pressure control, while the identified risk factors may inform targeted interventions.

## Introduction

High blood pressure is a major modifiable risk factor for cardiovascular diseases (CVD), contributing to about 7 million deaths worldwide [1]. Understanding differences among individuals in how blood pressure changes over time can inform tailored prevention and treatment strategies. However, previous studies examining average changes in blood pressure with age at population level, or subgroups of individuals exhibiting distinct trajectories of blood pressure, all have confirmed the conclusion that systolic blood pressure (SBP) rises with age [2,3,4,5,6,7,8,9,10,11,12,13]. Nevertheless, all these studies sought to identify blood pressure patterns for overall sample without regard to baseline blood pressure level and blood pressure patterns thus identified might be largely driven by differences in baseline blood pressure [4,5,8]. We postulate that heterogeneity in baseline blood pressure may have prevented these studies from identifying more nuanced yet clinically important trajectory patterns, such as individuals who experience improved blood pressure over time.

Moreover, the studies available so far assessing population heterogeneity in blood pressure trajectory have mostly focused on middle-aged and older adults, while opportunities for prevention and early treatment are prime in young adulthood [2,4,6,7,8]. We are only aware of two recent published studies that have characterized courses of blood pressure in early adulthood that all displayed various degrees of increasing trends [5,14]. The finding on associated risk factors is still limited and warrants further investigation.

Furthermore, it is of interest to know whether findings from western developed countries can be generalized to other populations, such as China where there is markedly different lifestyle, diet and culture, remains largely unknown. This paper studies Chinese workers because of growing prevalence of high blood pressure in China (increasing from 13.6% in 1991 to 18.0% in 2002 and to 25.2% in 2012) and the age-standardized prevalence of hypertension increasing by 1.4% per year, yet the rate of controlled blood pressure is low [15,16,17,18,19]. Moreover, the incidence of hypertension increased considerably in Chinese young adults from 1991–1997 to 2004–2009 and young adults with hypertension had lower rates of antihypertensive treatment and control compared with their older counterparts [11].

To address these knowledge gaps, we analyzed data from the Qingdao Port Cardiovascular Health Study in China to characterize heterogeneity of SBP trajectories in young adults [20]. We also assessed the association of individuals’ sociodemographic and baseline health characteristics with SBP trajectories. Findings from this study will facilitate more personalized prevention and treatment strategies targeting young individuals to help optimize their long-term blood pressure profiles.

## Methods

### Data Source and Study Population

The Qingdao Port Cardiovascular Health Study (QPCHS) is a prospective study of employees of the Qingdao Port Company, one of the largest companies in China specializing in international trade and ocean shipping with over 30,000 employees [20]. The QPCHS started in year 2000 with the goal of evaluating risk factors of chronic diseases and associated outcomes. It encompasses annual data collection on all participating employees via administered questionnaires and physical examinations.

At each physical examination, height, weight, waist circumference, resting heart rate, blood pressure, fasting blood glucose, total cholesterol, triglycerides and plasma uric acid were measured. The administered questionnaire ascertained information about demographics, socioeconomic status, lifestyle, personal health status, and family history of CVD including coronary heart disease, hypertension, and stroke from a parent or a sibling.

Participation in the study is voluntary. In its inception year (2000), 11262 individuals enrolled in the study. Additional employees participated in later years, resulting in a total sample of 20460 unique individuals aged 18–81 years during the 2000–2011 study period. Each participant was followed annually after their initial enrollment with up to 12 observations from 2000 through 2011. In this paper, we focused on the 12468 young individuals who were 18–35 years old at their first visit. They had an average of 4.6 observations (median = 3.0). All participants gave informed consent.

### Measures

The outcome measure was SBP. At each assessment, SBP was measured twice by a trained nurse and the average reading was used in analysis. To minimize the influence of extreme values and potential data error, SBP below 80 mmHg or above 230 mmHg were winsorized at 80 and 230 mmHg respectively (total of 48 measurements met these criteria).

Personal characteristics were defined at baseline. They included age (in years), gender (male versus female), education attainment (completed at least high school versus not), marital status (married versus not married), body mass index (BMI; kg/m^2^), waist circumference (inches), diet (whether consuming vegetables or fruits on ≥ 4 days of a week), smoking status (past or current smoker versus never smoked), hyperlipidemia (high low-density lipoprotein, high total cholesterol or high triglycerides versus none of these conditions), personal history of CVD (ever had myocardial infarction as shown on electrocardiogram, stroke and coronary heart disease versus never), diabetes (yes/no), use of anti-hypertensive medication (yes/no), and family history of CVD (i.e., history of hypertension, stroke or coronary heart disease from any of the parents or siblings versus none).

### Group-Based Trajectory Analysis

We used a group-based trajectory model to analyze changes in SBP over time [21]. The analysis is also called growth mixture model or latent class model and assumes that individuals cluster into distinct groups exhibiting different patterns of evolution trajectories for measure of interest. This approach allows the identification of distinct groups of individuals who share a similar underlying trajectory (latent class). Trajectory in this paper referred to the repeated measures of SBP throughout the observation period (up to twelve years), and we referred to the identified groups as trajectory groups. For each individual, the year when he/she first enrolled in the study was considered his/her baseline, and time was measured as years since baseline. We modeled the SBP trajectory as a function of time up to cubic term. One advantage of the method is its capacity to accommodate different numbers of repeated measures among different individuals, assuming the varying number is not directly related to the SBP. Therefore, individuals with at least one observation could be included in the analysis. We operationalized this analysis using a SAS macro PROC TRAJ [21].

We used the Bayesian Information Criteria (BIC) to guide our selection of the optimal number of trajectory groups [22]. Specifically, we calculated the BIC scores for each model with the number of trajectory groups from 2 to 8. The number of groups that generated the smallest BIC was considered as the optimal choice. In situations where the BIC kept improving with increasing number of groups, we determined the optimal model empirically by plotting BIC against the number of groups and selecting the group number when the BIC started to level off. Once the model with optimal number of groups is identified, posterior probabilities of membership in each of the groups were calculated for each individual, who was then assigned to the group for which s/he had the maximum posterior probability among all groups.

We stratified the overall sample based on participants’ baseline SBP level and performed a group-based trajectory analyses separately for each stratum. The cutoff values of SBP used for dividing the strata were determined based on the consideration of clinically guidance set by ACC/AHA SBP categories and the recent literature on the SBP risk categories for risk of metabolic syndrome [23,24]. According to the new ACC/AHA SBP categories, SBP of 130 or above is deemed hypertension. For male and female between age 31 and 35 years, ROC (receiver operating characteristic) study showed that the optimal cutoff is 114.5 and 110.5 respectively for the risk of metabolic syndrome [23,24]. So, we chose the first cutoff value for the baseline SBP at 110 mmHg and the second one at 130 mmHg. The first stratum included persons with SBP less than or equal to 110 mmHg at baseline without initial concern for high blood pressure, the second stratum included those with SBP above 110 but less than or equal to 130 mmHg as these participants may likely show some transition to above normal SBP during the follow-up period, and the third stratum included those with SBP above 130 mmHg which was deemed to be above the normal range for the age group.

### Personal Characteristics Associated with Distinct SBP Groups

At the end of group-based trajectory analysis, each person was assigned membership with a distinct SBP trajectory group. We numerically label a trajectory group according to the average SBP of that group with the group with lowest average SBP being labeled as first group. We examined the association of various personal characteristics with his/her trajectory group membership using ordinal logistic regression. Membership with ordinal label for a distinct trajectory groups was the dependent variable and personal characteristics were explanatory variables. The ordinal logistic regression models the cumulative probability of an ordinal category and all the categories with lower numerical label, so the categories of memberships have their own intercepts that are monotone increasing with their numerical label. All the ordinal categories by default share the same coefficients for the explanatory variables, which assumes an explanatory variable has equal association with each ordinal category of the trajectory membership. The default setting can be relaxed by including the interaction terms of a covariate and the dummy variables of the ordinal label to allow the covariate has different effects across different ordinal categories. The interaction effects can be assessed with likelihood ratio test.

We first assessed bivariate association by only including one personal characteristic at a time, and then examined multivariable association by adjusting for all personal characteristics simultaneously.

## Results

### Sample Characteristics

Baseline characteristics of the study sample are shown in Table 1. Overall mean SBP at baseline was 116.2 (standard deviation [SD] = 13.4), with 42.2% of the sample having a baseline SBP below or equal to 110 mmHg, 47.0% having a baseline SBP of 111–130 mmHg, and 10.8% have a baseline SBP greater than 130 mmHg.

Participants’ mean age was 26.3 (SD = 4.7) and most (83.5%) were male. Their mean BMI was 23.1 (SD = 3.7). Only 0.17% of the sample reported having a heart disease, while 20.4% had hyperlipidemia, and 19.1% had a family history of CVD. Across the strata, all these variables were statistically significant indicating individuals in different strata had quite different personal characteristics in health (Table 1).

**Table 1:** Sample baseline characteristics.

Baseline Variable	All (N = 12468)	Stratum 1 (N = 5257)	Stratum 2 (N = 5861)	Stratum 3 (N = 1350)	p-value

Age (year)	26.4 (4.7)	26.0 (4.6)	26.7 (4.7)	26.2 (4.9)	<0.0001
Male %	83.7	70.9	92.2	97.1	<0.0001
High school graduation %	38.8	41.3	38.5	30.4	<0.0001
Married %	26.4	26.3	26.7	26.0	0.874
Family history of CVD %	19.1	18.0	19.8	24.4	<0.0001
BMI	23.1 (3.7)	21.8 (3.2)	23.7 (3.5)	25.5 (4.1)	<0.0001
Waist circumference (inch)	31.4 (4.8)	29.8 (3.6)	29.8 (3.6)	29.8 (3.6)	<0.0001
High fruit/vegetable intake %	17.9	18.1	18.2	16.0	0.284
SBP	116.2 (13.4)	104.1 (6.6)	121.3 (5.1)	140.7 (8.9)	<0.0001
DBP	75.3 (9.8)	69.4 (7.1)	77.9 (7.8)	86.5 (11.3)	<0.0001
PP	40.9 (9.8)	34.7 (6.7)	43.3 (7.5)	54.2 (10.5)	<0.0001
Smoking %	29.7	25.8	32.6	32.7	<0.0001
Having diabetes %	0.10	0.04	0.12	0.30	0.0122
Having hyperlipidemia %	20.4	14.4	22.8	33.6	<0.0001
Having heart disease %	0.17	0.04	0.19	0.59	<0.0001
Anti-hypertensive medication	0.46	0.02	0.26	3.04	<0.0001

* Continuous variables are presented as Mean (Standard Deviation). Categorical variables are presented as proportion.

### SBP Trajectory Patterns

**Figure 1:**
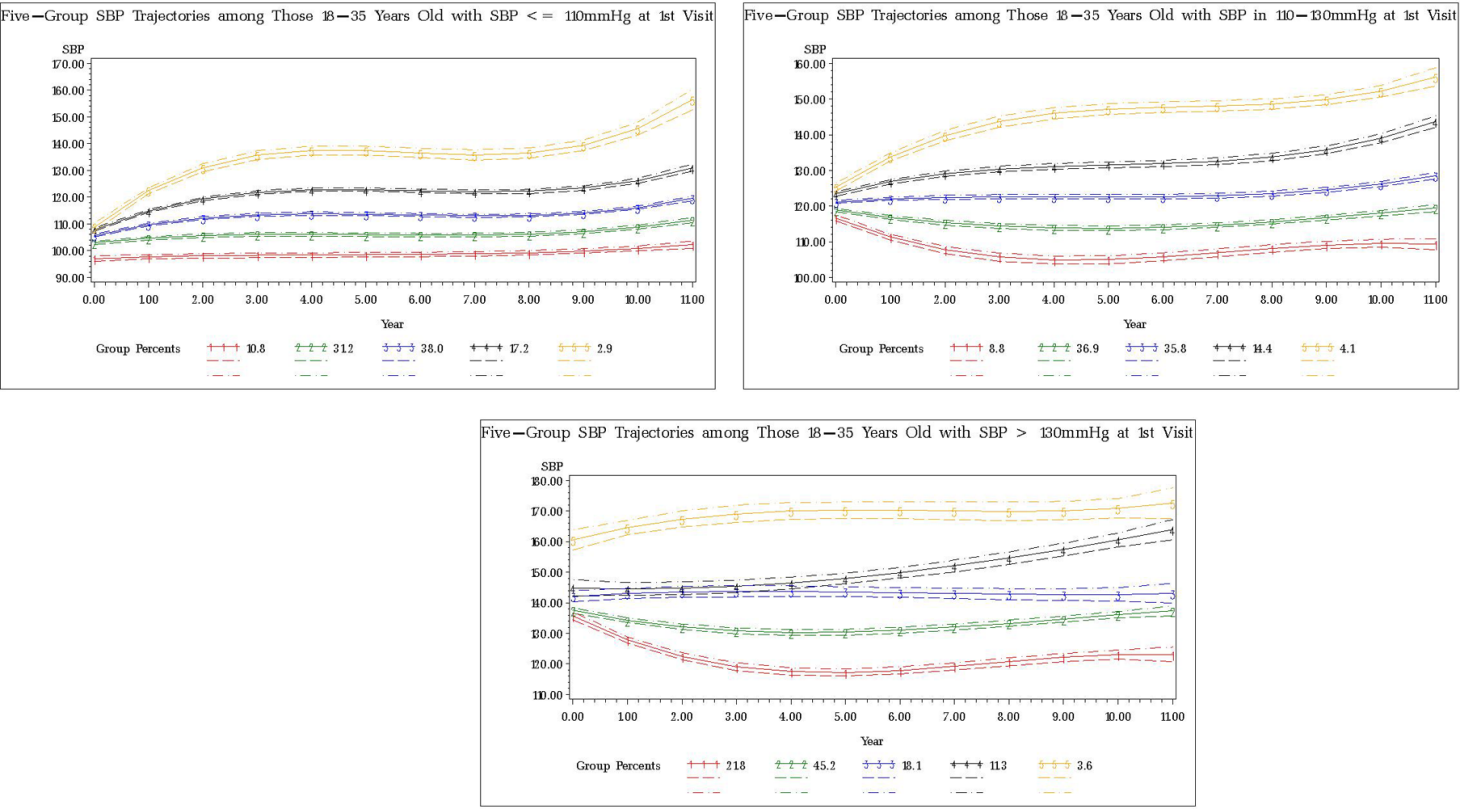
Trajectories of SBP Groups in Three Strata.

For all three strata, five groups emerged as the optimal number of SBP trajectories (Figure 1). Although the BIC values continued to improve with increasing number of groups, they improved at slower pace with greater than five groups (Appendix A). Within each stratum, trajectories were labeled as groups 1–5 based on their relative order of average SBP over time. In general, the trajectory groups within stratum showed a divergent pattern as they all started at a similar SBP level but ended with quite different levels.

For Stratum 1 (i.e., baseline SBP <=110 mmHg), group 1 had almost no change in SBP over time (remained at about 100 mmHg throughout the twelve years), groups 2–4 had slight increases in SBP (from 100–110 mmHg to 110–120 mmHg), yet group 5 experienced a substantial increase in SBP (from 110 to 150 mmHg) and was the only group in this stratum that ended up with SBP in the unhealthy range. Group 3 was the largest group in size accounting for 38.0% of individuals while group 5 was the smallest group accounting for 2.9% of the individuals.

For Stratum 2 (i.e., baseline SBP between 110 and 130 mmHg), group 1 showed a slight decrease in SBP over time (from about 115–120 to 110mmHg), while groups 2 and 3 showed almost no change in SBP over the years. In contrast, group 4 had a moderate increase in SBP (from 120 to 140 mmHg) and group 5 showed a more dramatic increase (from 125 to 155 mmHg), both reaching the threshold of hypertension towards later years of follow-up. Group 2 was the largest group accounting for 36.9% of the individuals and group 5 was the smallest group accounting for 4.1%.

For Stratum 3 (i.e., baseline SBP greater than 130 mmHg), everyone started with an SBP above the healthy range, but Group 1 demonstrated a “cured” pattern such that SBP decreased to below the level of hypertension at the end of the twelve years (from 135 to 120 mmHg) while SBP in the four upper groups remained above 130 mmHg. Groups 2 and 3 showed almost no change in SBP over time (stayed at about 140 mmHg), while group 4 showed steady increase in SBP (from 145 to 160 mmHg) and group 5 persistently stayed at high level of SBP (about 160–170 mmHg). Group 2 was the largest group accounting 45.2% of its individuals, and group 5 was the smallest groups accounting for 3.6% of its individuals.

**Figure 2:**
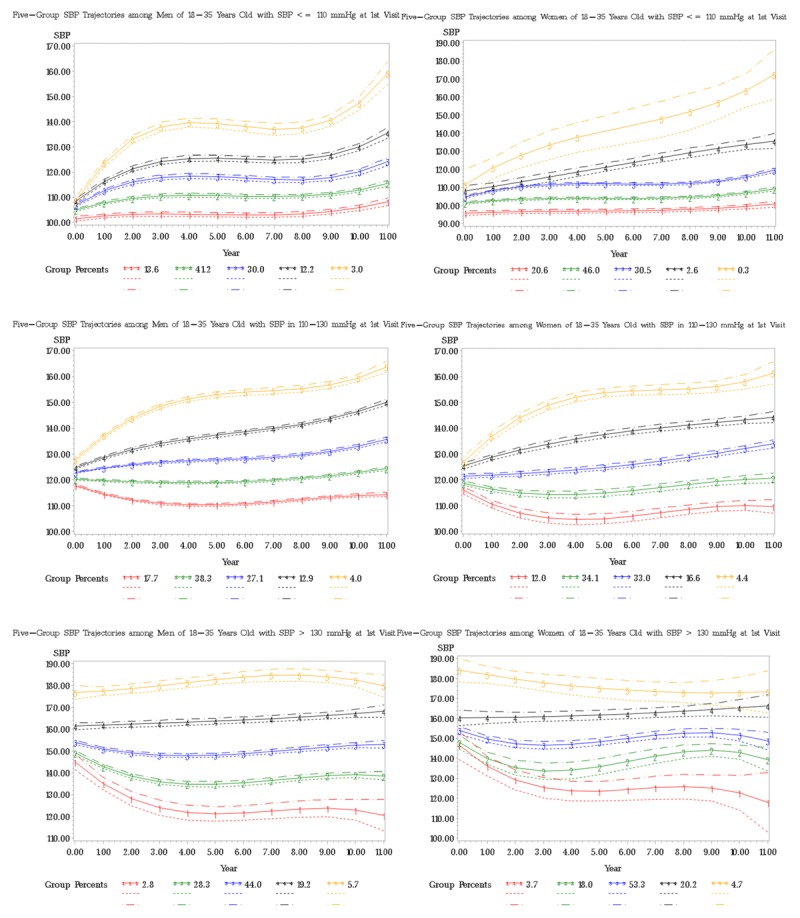
Sex-Specific Trajectories of SBP Groups in Three Strata.

Within each stratum of baseline SBP, we also obtained five groups of trajectories separately for men and for women (Figure 2), i.e., we conducted the trajectory analysis stratified on both baseline SBP and gender. The five trajectories between men and women look similar in trend, shape and overall range of each trajectory. So we focus on studying the association of personal characteristics using the trajectories from the combined men and women.

### Personal Characteristics Associated with the Distinct SBP Trajectory Groups

The association of personal characteristics with the distinct trajectory groups in each stratum are reported in Table 2. As a sensitivity analysis and also initially we did multinomial logistic regression, and found that the 95% confidence intervals of the estimates for the most of the same covariates overlapped across the difference groups. We presented the results from the ordinal logistic regression in the paper. We also tested for the interaction terms between the group label and each of the covariates in the ordinal logistic regression and only one interaction is significant at alpha level of 0.05.

**Table 2:** Association of participants’ baseline characteristics with the distinct SBP groups.

Patient baseline characteristics	Group 1	Group 2	Group 3	Group 4	Group 5	Bivariate p-value	Adjusted p-value	Adjusted OR

	***The Stratum with initial SBP ≤ 110 mmHg, Total N = 5229***							
Number of individuals	406	1536	2536	683	96			
Age (year)	25.9	26.0	25.8	26.3	25.8	0.716	0.0002	1.14**
Male sex %	23.4	58.2	78.7	95.2	95.8	<0.0001	<0.0001	5.18
High school graduation %	65.5	51.2	33.0	38.0	22.9	<0.0001	<0.0001	0.50
Married %	40.6	30.7	21.9	24.9	19.8	<0.0001	0.499	0.94
Family history of CVD %	23.7	17.6	16.6	20.2	22.9	0.580	0.0113	1.33
BMI	20.0	21.2	22.0	23.3	24.5	<0.0001	<0.0001	1.14
Waist circumference (inch)	26.7	28.6	30.3	31.8	33.0	<0.0001	<0.0001	1.18
High fruit/vegetable intake %	36.2	26.3	18.3	20.5	12.5	<0.0001	0.457	1.06
Baseline SBP (mmHg)	95.3	101.8	105.8	107.5	108.2	<0.0001	<0.0001	2.03**
Smoking %	7.1	23.7	28.4	32.2	24.0	<0.0001	0.0662	0.88
Having hyperlipidemia %	8.6	10.7	15.1	21.8	30.2	<0.0001	0.0101	1.68
Having heart disease %	0	0	0.08	0	0	0.651	0.703	1.68
Having diabetes %	0	0	0.08	0	0	0.651	0.534	2.31
Anti-hypertensive medication	0	0	0.04	0	0	0.749	0.973	0.94
	***The Stratum with initial SBP in 110–130 mmHg, Total N = 5861***							
Number of Individuals	284	2538	2343	531	165			
Age (year)	26.7	26.3	26.7	27.9	27.2	<0.0001	<0.0001	1.13**
Male sex %	76.4	89.9	94.9	97.9	97.0	<0.0001	<0.0001	3.22
High school graduation %	73.2	36.1	35.1	48.0	33.9	0.0028	<0.0001	0.65
Married %	41.2	23.0	25.7	39.4	29.7	0.0010	0.0013	1.29
Family history of CVD %	21.4	16.4	20.6	29.4	27.3	<0.0001	<0.0001	1.52
BMI	21.6	23.0	24.2	25.3	25.4	<0.0001	<0.0001	1.14
Waist circumference (inch)	29.5	31.4	32.7	34.0	34.1	<0.0001	<0.0001	1.12
High fruit/vegetable intake %	44.0	20.7	21.2	28.4	16.3	0.0108	0.0182	0.84
Baseline SBP	117.4	119.0	123.1	124.8	125.7	<0.0001	<0.0001	2.69**
Smoking %	30.6	30.0	33.3	42.2	35.8	<0.0001	0.339	1.06
	***The Stratum with initial SBP in 110–130 mmHg, Total N = 5861***							
Having hyperlipidemia %	15.8	18.3	24.9	34.3	38.2	<0.0001	<0.0001	1.64
Having heart disease %	0	0.24	0.13	0.19	0.61	0.958	0.936	0.96
Having diabetes %	0	0.08	0.13	0.38	0	0.192	0.323	2.01
Anti-hypertensive medication	0	0.12	0.34	0.75	0	0.0248	0.0409	2.68
	***The Stratum with initial SBP > 130 mmHg, Total N = 1350***							
Number of Individuals	156	932	143	64	55			
Age (year)	27.6	26.6	29.0	29.7	27.7	<0.0001	0.0380	1.16**
Male sex %	98.7	96.5	99.3	98.4	96.4	0.782	0.636	1.17
High school graduation %	55.1	22.5	43.4	68.8	14.5	0.788	0.0005	0.58
Married %	40.4	18.1	42.7	70.3	23.6	0.0002	0.0196	1.53
Family history of CVD %	32.1	18.1	39.2	54.7	34.6	<0.0001	0.0002	1.721
BMI	24.7	25.3	26.7	26.7	27.6	<0.0001	<0.0001	1.08
Waist circumference (inch)	33.0	33.9	35.6	35.3	35.7	<0.0001	<0.0001	1.08
High fruit/vegetable intake %	39.7	12.7	30.1	48.4	20.0	0.267	0.871	1.03
Baseline SBP	137.3	138.9	143.7	149.4	163.6	<0.0001	<0.0001	1.05**
Smoking %	38.5	28.8	39.2	59.4	34.5	0.0285	0.669	1.06
Having hyperlipidemia %	35.3	28.8	50.3	56.2	40.0	<0.0001	0.0077	1.41
Having heart disease %	0.64	0.43	1.40	0	1.82	0.339	0.321	2.04
Having diabetes %	0	0.32	0	1.56	0	0.505	0.767	1.35
Anti-hypertensive medication	1.9	2.4	4.2	4.7	12.7	0.0006	0.0038	2.47

** Adjusted odds ratio (OR) for baseline age and baseline SBP was calculated based on 5 year and 5 mmHg increment, respectively.

Multivariable ordinal logistic regression analysis showed that in all strata, advanced age, male sex, not graduating from high school, a family history of CVD, having greater BMI, having greater waist circumference, and presence of hyperlipidemia were each consistently associated with a significantly higher likelihood of a person following a trajectory of higher SBP (p < 0.05 for all). While being a past or current smoker significantly increased a person’s risk of having higher SBP trajectories only in unadjusted analysis.

Other personal characteristics were only associated with trajectory group membership in selected strata. Not being married and taking anti-hypertensive medication at baseline were significantly associated with trajectory groups with higher SBP in the 110 < SBP ≤ 130 mmHg and SBP ≥130 mmHg strata (p < 0.05 for all). Male sex was associated with an increased odds of having trajectories of higher SBP in the SBP ≤ 110 mmHg and 110 < SBP £130 mmHg strata (p < 0.001 for all). Consuming high fruit and vegetable diet was associated with a significantly decreased likelihood of having a trajectory of lower SBP in the stratum with 110 < initial SBP ≤ 130 mmHg (adjusted OR = 0.84, p = 0.02).

Noticeably, three groups exhibited particularly unique trajectory patterns. Group 5 in the SBP ≤ 110 mmHg stratum was the only group in this relatively healthy stratum that experienced dramatic increase in SBP. Further examination comparing personal characteristics in this group versus other groups in the stratum with multivariate binary logistic regression revealed that a statistically significant lower proportion of individuals in this group had completed high school education and a higher proportion of individuals had family history of CVD, being hyperlipidemia, and being past or current smoker. The group also had significantly higher BMI and larger waist circumference than the other groups at baseline. In contrast, Group 1 in the SBP ≥130 mmHg stratum was the only group in this relatively unhealthy stratum that achieved substantial improvement in blood pressure. Further examination revealed that a statistically significant higher proportion of individuals in this group having high school education, significantly lower BMI and smaller waist circumference than the other groups. Group 4 in the SBP ≥130 mmHg stratum, another noticeable group, despite having highest proportion of being married and fruit/vegetable intake, and 2nd highest proportion of high school graduation among all groups across the strata, had the 2nd highest SBP trajectories with a steady increase of SBP over time. The group had highest proportion of the family history of CVD and hyperlipidemia, which may explain the ‘noxious’ trajectory of SBP in this group.

## Discussion

Our study extends the current literature in several important ways. We identified diverging patterns of SBP trajectories among individuals who had similar SBP at baseline, particularly we uncovered a group of individuals whose blood pressure would improve over time. Prior studies typically used an ‘all-inclusive’ approach to identify SBP trajectory patterns for the overall sample without regard to baseline blood pressure level [4,5,8]. As a result, their identified different trajectory patterns may be largely driven by differences in baseline blood pressure and only broad patterns can be detected. This might mask smaller yet clinically important trajectory patterns. In contrast, by closely examining individuals with similar SBP at baseline, we were better able to identify more intricate differences in how their SBP evolved over time.

It is possible that subjects in different strata may have similar trajectories except the baseline SBP. For example, the SBP trajectory in Group 4 of Stratum 1 is similar to that in Group 3 of Stratum 2, both trajectories were around SBP of 120 mmHg. Moreover, the two groups had strikingly similar distribution of patient characteristics in terms of age, sex, % high scholl graduation, % married, % family history of CVD, waist circumference, fruit/vegetable intake, % smoking and % hyperlipidemia. However, other trajectories are not similar across different strata.

We provided important findings on heterogeneity of SBP trajectory in a young Chinese population. A recent study on life course blood pressure discovered that women exhibited a steeper increase in BP starting in the third decade [25]. We did not find sex differences in terms of trend and range of each trajectory by further stratifying on gender, probably because our study only included adults younger than 35 years old. Our focus on younger adults provides unique data to inform prevention efforts. Heterogeneity in their SBP path highlights the importance and potential feasibility of tailoring public health interventions and clinical care to better address individuals’ needs in maintaining healthy blood pressure. Our study found that individuals with less than high school education, greater BMI, and greater waist circumference at baseline were more likely to have a poor SBP trajectory. These findings are generally consistent with previous literature in the U.S. and European countries [26,27]. In particular, we observed individuals who were able to recover from elevated blood pressure and relatively healthy young adults who experienced a more rapid increase in blood pressure leading to hypertension. The personal characteristics in these two groups that were significantly different from the other groups within their respective strata were education attainment, BMI and waist circumference. These unique SBP trajectory patterns deserve closer clinical and research attention. The former group suggests that there are opportunities to effectively control blood pressure, while the latter group highlight the importance of prevention among healthy young adults [28]. Further research to more closely examine the experience of these two distinctive subgroups would help inform more targeted interventions and improve efficiency and effectiveness in hypertension prevention and treatment.

The identification of the ‘noxious’ Group 4 in the SBP≥130 mmHg stratum also indicated that certain family history may confer upon individual high risk of hypertension in spite of healthy lifestyle choice. Individuals in the group had several lifestyle variables in marital status and diet associated with prevention of hypertension, nevertheless still had persistent high blood pressure with steady increase over time. The identification of the group suggested the possible presence of nonmodifiable risk factors such as some genetic factors for hypertension as the group had highest proportion with family history of CVD. These findings highlight the need for tailored prevention and treatment interventions.

Our study has several limitations. First, the QPCHS is a geographically restricted cohort and is composed of employees from a particular industry, hence our findings may not be generalizable to other populations. Nevertheless, our stratified group analysis provided an effective approach that may help identify unusual subgroups of patients warranting particular attention or informing future care. Furthermore, our finding may shed light on potential trajectory patterns in other developing countries. Second, although the QPCHS gathered personal information on many important parameters that may influence blood pressure. We did collect personal information on exercise and type of occupational work (manual versus desk) which may also influence blood pressure, however, there were too many missing data to prevent us from conducting analysis related to these personal characteristics. Also, future research exploring the potential role of medication in modifying SBP trajectories will be informative. Third, we used standard office/clinic blood pressure measurements which might have caused the ‘white-coat-effect’ which biases the SBP towards higher level, instead an automated office device may be able to obtain a systolic blood pressure that correlates more accurately with true cardiovascular risk. Lastly, we used a data-driven approach to identify latent trajectory groups. Development of methodologies allowing for validation of the empirically identified trajectory patterns will help assess the usefulness of this line of research in guiding clinical care.

## Appendix

BIC plots by number of trajectory groups in each of the three strata.
